# Spatiotemporal Bayesian estimation of the number of under-reported COVID-19 cases in Victoria Australia

**DOI:** 10.7717/peerj.14184

**Published:** 2022-10-21

**Authors:** Dinah Jane Lope, Haydar Demirhan

**Affiliations:** Mathematical Sciences Discipline/School of Science, RMIT University, Melbourne, Victoria, Australia

**Keywords:** Under-reported cases, Incidence, Detection probability, Robust logistic regression, Bayesian model averaging, Poisson regression, Logit model, Trend models

## Abstract

Having an estimate of the number of under-reported cases is crucial in determining the true burden of a disease. In the COVID-19 pandemic, there is a great need to quantify the true disease burden by capturing the true incidence rate to establish appropriate measures and strategies to combat the disease. This study investigates the under-reporting of COVID-19 cases in Victoria, Australia, during the third wave of the pandemic as a result of variation in geographic area and time. It is aimed to determine potential under-reported areas and generate the true picture of the disease in terms of the number of cases. A two-tiered Bayesian hierarchical model approach is employed to estimate the true incidence and detection rates through Bayesian model averaging. The proposed model goes beyond testing inequality across areas by looking into other covariates such as weather, vaccination rates, and access to vaccination and testing centres, including interactions and variations between space and time. This model aims for parsimony yet allows a broader range of scope to capture the underlying dynamic of the reported COVID-19 cases. Moreover, it is a data-driven, flexible, and generalisable model to a global context such as cross-country estimation and across time points under strict pandemic conditions.

## Introduction

In late 2019, the unexpected surge of COVID-19 shook the world, causing health systems to collapse, and many people were passing away and in urgent need of health care services. The spread of this infectious disease developed at a fast and insurmountable pace where health systems struggled given their unpreparedness and limited resources. In addition, the limited information on this infectious disease made effective control and prevention more challenging to plan and implement. With this, researchers and scientists worldwide made an active and enormous effort to model and predict the rapid surge of the disease (De Oliveira et al., 2020; Jewell, 2021).

Investigating the issue of under-reporting in disease surveillance and notification is essential to accurately and timely evaluate disease burden by looking at better estimates for mortality in addition to morbidity rates (Kupek, 2021; Albani et al., 2021) and implement mitigation measures as swiftly as possible. Lau et al. (2021) mentioned that estimates for under-reporting rate can be recorded through testing. However, given the lockdowns and limited movement brought about by the menace of getting infected, implementing area-wide testing seems implausible. Testing is an important driver in detecting the daily cases of COVID-19. When people do not move forward to get tested, it makes it harder to reflect the real trend or spread of the disease in the community in the daily figures (Rader et al., 2020). On the other hand, other factors can impact the increase or decrease in the daily testing trend. There is a possibility that trend is a result of time and area-related factors such as cold weather or the winter season in which more people can experience flu-like symptoms (Moriña et al., 2021). In addition, the availability of testing centres or healthcare services can impact the accessibility to the community to get tested (Rader et al., 2020; Mena et al., 2021; Lau et al., 2021). In that sense, inequality in accessing health services can be influential on the number of unreported cases. With these, the incorporation of various conditions that can impact the number of daily cases of COVID-19 needs to be considered. There is a need for exploratory analysis in a broader scope of factors or covariates that can impact the dynamics of the spread and detection to generate better estimates of the disease burden.

### Spatial inequality

Several studies have explored the impact of spatial variations on the rise of COVID-19. In a study conducted by Rader et al. (2020) on geographic access differences in the United States relative to COVID-19 testing sites, results suggest that this disparity is associated with sociodemographic factors consequently linked to poor health access and outcomes. Similarly, Mena et al. (2021) analysed the incidence and mortality attributed to COVID-19 to understand spatial variations in disease burden in Chile. Results show higher infection fatality rates in lower-income municipalities, given their comorbidities and poor access to health care. Furthermore, this spatial inequality across areas is apparent in the delay and capacity of testing.

In another study in the United States, Figueroa et al. (2020) reviewed spatial inequality by looking at the communities’ economic, demographic, and occupational factors. Results suggest that it may help mitigate the spread of COVID-19 in minority communities by improving health care for foreign-born non-citizens, addressing crowded housing, and protecting food service workers. Relatively, Sy, White & Nichols (2021) found that populated areas increase the rates of contact leading to disease transmission of COVID-19. The study suggests that geographic differences should be considered in estimating the transmission rate for resource allocation and proper planning. Neighbouring areas also impact this geographic inequality of COVID-19 as studied by Martins-Filho et al. (2020). As recorded, mortality estimates in the North and West zones of Aracaju in Brazil are the highest where many socio-economically deprived neighbourhoods are located. In a similar study by Bilal et al. (2021) in the cities in the United States: New York, Philadelphia, and Chicago, lower testing rates and higher positive ratios, mortality rates and confirmed case rates are recorded in neighbourhoods with higher social vulnerability.

### Temporal inequality

Apart from the spatial differences, several studies explored the effect of temporal variation in data at every specific time point of the COVID-19 pandemic. This enables us to see and infer from the growth of the daily disease cases to improve planning and intervention and predict potential outbreaks. In Australia, Trauer et al. (2021) conducted a study in the second wave of COVID-19 on the aggressive policy interventions implemented in the Victorian state across time. To determine the reasons for the pandemic’s peak and decline in this second wave, they explored the effect of time-varying processes on their parameters, including testing rates, face coverings, physical distancing and population mobility. Results showed that these interventions greatly helped overturn the pandemic growth led by the use of face coverings. In addition, the study showed that the impact of these time-varying interventions could represent other behavioural changes.

During the earlier stages of the pandemic, Brandenburg (2020) attempted to model the growth of the pandemic using piece-wise quadratic behaviour relative to the changes in the population dynamic and interventions. Results showed that quadratic growth laws were mainly the result of partial safety measures and that the maximum possible infection levels have been attained with the existing safety measures. It is deemed that the quadratic behavior can be driven by surrounding growth when additional spreading occurs in the outskirts of an infected area. On the other hand, Li et al. (2021) explored the use of time-series models such as Holt exponential smoothing and autoregressive integrated moving average (ARIMA) models to predict the COVID-19 cases in China. Findings suggest that the recalibrated ARIMA model can detect the effects of the interventions and unusual changes in trends. This result is deemed beneficial in trend analysis of the pandemic and evaluating the interventions across time points.

The aforementioned studies demonstrated the importance of the spatial and temporal effects in understanding the spread of COVID-19. Moreso, these space and time variations played an important role in either increase or decrease of COVID-19. Lau et al. (2021) evaluated under-testing and under-reporting of COVID-19 cases in multiple global epicenters (which includes South Korea, Japan, China, Spain, France, Italy, Germany, Iran and the United States) and found that the differences in testing and overall health care and medical system resulted in significantly different COVID-19 cases and mortality rates, which further emphasize the apparent impact of inequality across countries.

These analyses have yet to explore an approach surrounding models that involve both spatial and temporal inequality in the model that estimates the under-reported cases of COVID-19. However, these methodologies provide an array of frameworks and a combination of covariates which will be considered in our study.

In this study, we aim to incorporate the spatial and temporal effects in investigating and inferring the number of under-reported cases of COVID-19. Estimating the number of under-reported cases is essential in disease surveillance to provide a better-informed picture of the spread of COVID-19. We utilized a two-component Bayesian model that investigates the case detection rate and incidence of COVID-19. In these two components, we incorporate covariates with variations in space and time which can impact the spread and rise of the daily disease cases. In our model, we considered not only the inequality in the health services access of people in the community but also the available access to them, such as the availability and number of testing and vaccination centres. These facilities aid the detection, reporting, and control of the disease. As an improvement to existing studies, the model from the studies of Shaweno et al. (2017), Stoner, Economou & Da Silva (2019) and Zipfel, Colizza & Bansal (2021) are improved. We added a *floating covariate* that captures the possible effect of the covariate on either of the two components in the model. Furthermore, the Bayesian Model Averaging (BMA) is employed to determine the categorization of this floating covariate. Lastly, we introduced the use of robust logistic regression to address the impact of outliers in the distribution of the disease as a new contribution over the previous forms of the approach by Stoner, Economou & Da Silva (2019) and Lope, Demirhan & Dolgun (2022). Mainly, this study aims to fill in the gap by (i) proposing a model that allows the inclusion of wide-scope covariates but preserves parsimony, (ii) allowing the interaction and model specification of the covariates in and between space and time and (iii) allowing the data to determine the effect of differences in space and time in the incidence and detection or reporting of the disease.

## Materials and Methods

Shaweno et al. (2017) and Stoner, Economou & Da Silva (2019) explored using a Bayesian technique to estimate tuberculosis occurrence in Ethiopia and Brazil, respectively. This model is composed of incidence rate and case detection rate models. With this approach, they were able to identify under-reported areas of tuberculosis. Furthermore, given the two-component modelling, it distinguishes the true incidence from the case detection rate, which helps to explore whether the increase or decrease of the reported cases is due to the health system’s performance. Findings show a significant difference in reported rates and estimated incidence rates in areas with no health facilities. In addition, this model was used further by Zipfel, Colizza & Bansal (2021) to explore the transmission and surveillance of influenza in the United States. Lope, Demirhan & Dolgun (2022) introduced the floating variable concept into this model and used Bayesian model averaging to model influenza in Victoria, Australia. Both studies identified areas with influenza hotspots that were mostly overlooked by conventional influenza surveillance and notification. The current study leveraged the approach from Shaweno et al. (2017); Stoner, Economou & Da Silva (2019) and Lope, Demirhan & Dolgun (2022) to estimate the effect of spatial and temporal inequality in disease detection which can lead to under-reporting.

We follow the assumption that individual cases are detected independently at a fixed rate and are conditional on individual incidence (Shaweno et al., 2017; Stoner, Economou & Da Silva, 2019). The two main component models of the Bayesian framework are incidence and case detection probability models, in which *t* shows the time index and *s* is the space index to encapsulate the temporal and spatial effects, respectively. The incidence component is defined as in Eq. (1):

(1) \begin{eqnarray*}{\text{Incidence}}_{t,s}\sim \text{Poisson}({\lambda }_{t,s}),\end{eqnarray*}

where the model for the mean incidence rate at time *t* and in area *s* is given in Eq. (2):

(2) \begin{eqnarray*}\log \nolimits ({\lambda }_{t,s})={\alpha }_{0}+\sum _{k=1}^{K}{\alpha }_{k}{\text{u}}_{t,s}^{(k)},\end{eqnarray*}

such that {*u*^(*k*)^} is the vector of covariates that induces the number of disease cases with the offsets such as population counts. The expected number of detected cases, Z_*t*,*s*_, follows a binomial distribution conditional on the hidden true incidence rate and the probability of the case being detected:

(3) \begin{eqnarray*}{\text{Z}}_{t,s}\sim \text{Binomial}({\pi }_{t,s},{\text{Incidence}}_{t,s}),\end{eqnarray*}

where the model for the case detection probability is defined in Eq. (4):

(4) \begin{eqnarray*}\log \nolimits \left( \frac{{\pi }_{t,s}}{1-{\pi }_{t,s}} \right) ={\beta }_{0}+\sum _{j=1}^{J}{\beta }_{j}{\text{v}}_{t,s}^{(j)}.\end{eqnarray*}

In Eq. (4), {v^(*j*)^} is the vector of covariates attributable to the disease detection process. This captures the intensity of under-reporting and the influence of the related covariates. By using integration and Bayes’ rule (Stoner, Economou & Da Silva, 2019), we have

(5) \begin{eqnarray*}{\text{Incidence}}_{t,s}-{\text{Z}}_{t,s}\sim \text{Poisson}((1-{\pi }_{t,s}){\lambda }_{t,s}).\end{eqnarray*}

For this model, the conditions to get reliable estimates of under-reported cases include (i) every individual case needs to have an equal chance of being independently reported, and (ii) the information in the observed data needs to be supplemented by additional information to distinguish under-reporting and the true incidence rate (Stoner, Economou & Da Silva, 2019). The first point is the main assumption for the model to statistically formulate the likelihood function. The chance of being reported for each individual may differ across the states. However, since we consider the cases by Local Government Areas(LGAs), every individual in each LGA has an equal chance of being reported. For the second point, we propose to use BMA to relax the need for supplementary information by giving a chance for covariates to appear in both case detection and incidence rate components of the model. The main limitation of this model is the need for observed cases to produce accurate estimates of the number of under-reported cases. In the case study of this article, we provide a detailed assessment of deviances, especially for zero and low numbers of observed cases, to ensure that the model does not produce unreasonably high deviances.

In this study, to improve the case detection probability model in Eq. (4) against outliers, we introduced the robust logistic regression by including a parameter *γ* such that

(6) \begin{eqnarray*}{\pi }_{t,s}=\gamma \cdot \frac{1}{2} +(1-\gamma )\cdot \text{logit} \left[ {\beta }_{0}+\sum _{j=1}^{J}{\beta }_{j}{\text{v}}_{t,s}^{(j)} \right] .\end{eqnarray*}

In Eq. (6), the logistic model becomes a mixture of sources; one from the covariates and the other source is mere randomness in which, let us say the *x* value is generated from flipping a fair coin: *x* ∼ Bernoulli(*μ* = 1/2). Then, every data point has a small probability (*γ*) of being generated by the random process, but usually, with probability (1 − *γ*) coming from the logistic function of the covariates. This approach helps reduce the slope of the estimated logistic curve to avoid getting data points with zero probability estimates (Kruschke, 2015, p. 635).

Furthermore, Stoner, Economou & Da Silva (2019) and Lope, Demirhan & Dolgun (2022) mentioned the challenge of categorising a covariate between the two models, especially when the covariate seems to impact both incidence and case detection rates. Lope, Demirhan & Dolgun (2022) establish a covariate called a *floating covariate*. This floating covariate aims to address this issue of ambiguity in categorisation. The introduction of floating covariates results in multiple models that can fit the data well. To capture the model uncertainty created by having floating covariates, we induce Bayesian model averaging (BMA) into the modelling framework. We create a model space ℘(*S*) in a way that all elements are given the opportunity to impact the parameter of interest Δ considering the data D (Hoeting et al., 1999). With such, the parameter of interest will have a posterior distribution formulated in Eq. (7),

(7) \begin{eqnarray*}P(\Delta {|}D)=\sum _{m\in \wp (S)}^{}P(\Delta {|}m,D)P(m{|}D).\end{eqnarray*}

BMA provides the flexibility to visit the models in a model space ℘(*S*) that is proportional to their respective posterior model probabilities calculated as such,

(8) \begin{eqnarray*}P(m{|}D)= \frac{P(D{|}m)P(m)}{\sum _{{m}^{{^{\prime}}}\in \wp (S)}^{}P(D{|}{m}^{{^{\prime}}})P({m}^{{^{\prime}}})} ,\end{eqnarray*}

where *m* is the model of interest and *m*′ shows each model in the model space.

## Case Study: Victorian COVID-19 Data

The study area is Victoria, Australia, around the time of the third wave of the pandemic in the state, which dates from 28 August 2021 to 8 November 2021, leading to 73 time points. The areas in Victoria are spatially aggregated into 79 LGAs. The Victorian data is sourced from the COVID LIVE website (https://covidlive.com.au/), which is verified by state and federal health departments. Population data is sourced from the Australian Bureau of Statistics (Australian Bureau of Statistics, 2022) whereas the climate data is taken from the Bureau of Meteorology (Australian Government—Bureau of Meteorology, 2021).

### Descriptive analysis

**Figure 1:**
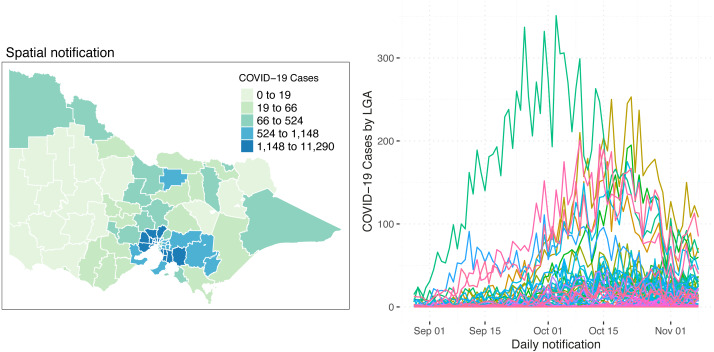
Spatial distribution (left) and the number (right) of reported COVID-19 cases in Victoria, Australia, from 28 August 2021 to 8 November 2021. On the right side of the diagram, each line represents an LGA.

Figure 1 shows the Spatio-temporal distribution of COVID-19 cases and the trends by LGA from 28 August 2021 to 8 November 2021. The left-hand side of Fig. 1 represents the spatial distribution of the total number of reported COVID-19 cases within the study time points. It shows that most of the reported cases are concentrated in metropolitan areas and slowly ease as it goes outward to the regional areas. In the western areas, a very small total of reported COVID-19 cases is observed as compared to the eastern areas. On the other hand, as shown on the right-hand side of the diagram, there are a number of LGAs where the daily trend is recognisably higher though most of the LGAs were running at the same trend and intensity. It is of interest to model the LGAs with different trend characteristics separately. Thus, we identified 8 LGAs with the most reported COVID-19 cases, namely Hume, Brimbank, Casey, Greater Dandenong, Melton, Moreland, Whittlesea and Wyndham. Nonetheless, a relatively small daily number of reported COVID-19 cases are seen in the state, ranging from 0 to 350 cases a day.

This study aims to cover a wide range of covariates that can explain the incidence rate and case detection probability of COVID-19 cases while maintaining a parsimonious model as much as possible. With this, after thoroughly exploring available data within the considered time span, the incidence is explained using the percentage of second vaccination rate, the number of vaccination centres and the balancing effect of the population counts. The structured and unstructured effect of the spatial area is also taken into account. On the other hand, the number of tests conducted, minimum temperature and direction of the wind were considered as the exploratory covariates for the case detection probability. The minimum temperature and wind direction are correlated with the rise of flu-like symptoms in the community. Northerly winds bring a significant amount of pollen to the study area and cause a notable increase in the number of people having flu-like symptoms, which has the potential to increase the number of people getting tested.

**Figure 2:**
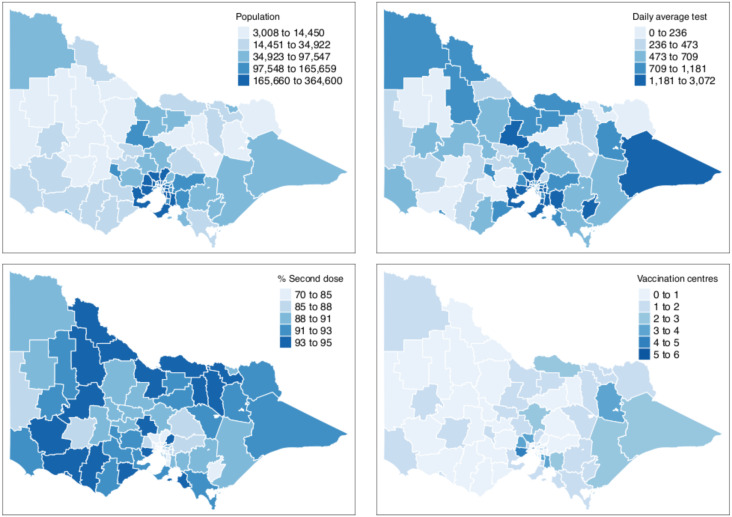
Distribution of covariates by LGA in Victoria, Australia. Bins for the population count, % second dose and daily average tests are equally divided into five breaks to show each 20th percentile bracket.

**Figure 3:**
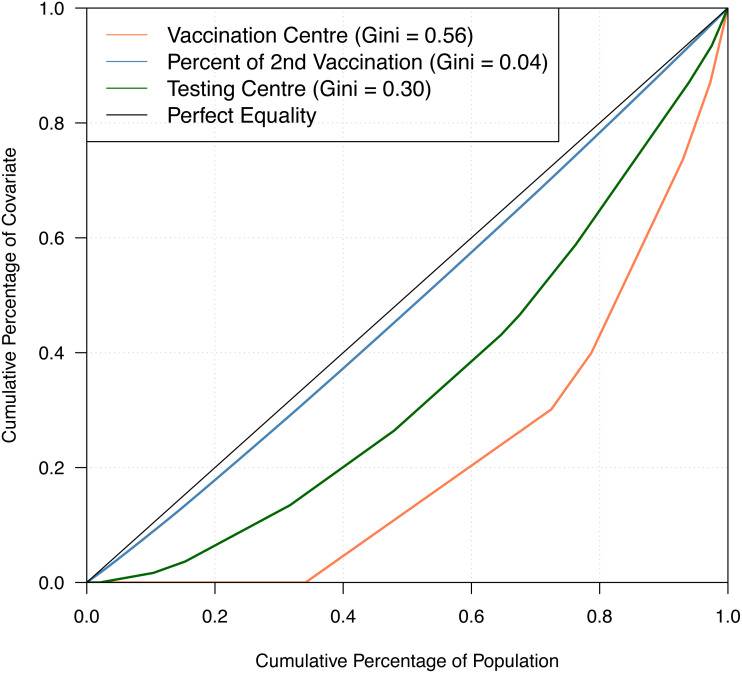
Gini coefficient and Lorenz curve.

Table 1 shows the summary statistics of the aforementioned covariates whilst Fig. 2 shows their spatial distribution, where each color corresponds to every 20th percentile of the covariates’ distribution. In Table 1, the average number of COVID-19 cases on a daily basis across areas is very small, roughly 0.02 per cent of the average population and 2 per cent of the average daily tests. The study time points are recorded to have a relatively cold minimum temperature at 10 °C and experienced close to half gust of north wind as it is spring season in Australia. For the minimum temperature and direction of the wind, the values for these covariates were the same across all LGAs, varying only on a daily basis. This is because we would expect a minimal variation across the very small and compact LGAs of the state given. In Fig. 2, it is observed that the areas with a high percentage of second vaccination dose are the areas with a lesser population; thus, it was quickly saturated. On the other hand, unsurprisingly, in relation to Fig. 1, denser areas in the metropolitan region recorded a higher total number of reported COVID-19 cases. In addition, these denser areas have more tests conducted and strategically have a higher number of vaccination centres in place. To better understand the state’s inequality of health resources and services, Fig. 3 shows the distribution of vaccination and testing centres in addition to the percentage of second vaccination using the Gini coefficient and Lorenz curve. The vaccination centres are relatively not equally distributed across areas with Gini = 0.56, wherein the closer the value of Gini to 0 means more equality in distribution. However, these vaccination centres seemed to have been strategically allocated where densely populated areas have a higher number of vaccination centres, and the state achieved an almost equal percentage of second vaccination as shown in Fig. 2. On the other hand, testing centres are relatively equal across with Gini = 0.30, where 80 per cent of the population shares around 65 per cent of testing centres in the state.

**Table 1:** Covariates’ summary statistics across space and time.

Covariates	Mean	Median	SD
COVID-19 cases	13	1	32
Population count	84,757	47,725	80,558
Number of daily tests	769	644	643
Second vaccination (%)	60.1	58.1	17.6
Minimum temperature (°C)	10.1	10.0	3.1
Max wind direction (binary, north-referenced): 44% of the observations are north-wind			

### Bayesian modelling and model space

Applying the Bayesian framework of this study using the Victorian COVID-19 dataset, the true incidence and case detection probability are implemented through Eqs. (1), (2), (3), (4) and (5). For the incidence component in Eq. (1), we establish the model in Eq. (9):

(9) \begin{eqnarray*}\log \nolimits ({\lambda }_{t,s})={\text{pop}}_{s}+{\alpha }_{0}+{\alpha }_{1}{\text{secondvac}}_{t,s}+{\alpha }_{2}{\text{vaccentres}}_{s}+{\phi }_{s}+{\theta }_{s}+I(\cdot )f({t}_{\lambda }),\end{eqnarray*}

where *f*(*t*_*λ*_) is a function of time defined as *f*(*t*_*λ*_)= *α*_3_*t* + *α*_4_*t*^2^ + *α*_5_*t*^3^ and is treated as a floating covariate controlled by the indicator function *I*(⋅), likewise in Eq. (10). As seen in the trend patterns in Fig. 1, there were eight LGAs that had a greater increase in daily reported cases of COVID-19 and showed a distinct trend as compared to the remaining LGAs. Therefore, we isolated those 8 LGAs and added the time function to the model to capture the drastic trend. The covariate *pop* represents the population count and is treated as a balancing effect, *secondvac* shows the percentage of the second vaccination, and *vaccentres* shows the number of vaccination centres of the area *s*. Considering underlying spatial characteristics, *ϕ*_*s*_ represents the structured spatial effect while *θ*_*s*_ is the spatial unstructured random effect. *ϕ*_*s*_ is modelled as an intrinsic Gaussian autoregressive model (Besag, York & Mollié, 1991) to capture the effects of neighbouring areas wherein areas are considered neighbours when *s*′ ≠ *s* shares a common spatial boundary.

The expected number of detected cases, Z_*t*,*s*_, follows a binomial distribution conditional on the hidden true incidence rate and the probability of the case being reported or detected as given in Eq. (3) where we define Incidence_*t*,*s*_ as in Eqs. (1) and (9), and set the model in Eq. (10) for the case detection probability:

(10) \begin{eqnarray*}\log \nolimits \left( \frac{{\pi }_{t,s}}{1-{\pi }_{t,s}} \right) ={\beta }_{0}+{\beta }_{1}{\text{tested}}_{t,s}+{\beta }_{2}{\text{mintemp}}_{t}+{\beta }_{3}{\text{northwind}}_{t}+I(\cdot )f({t}_{\pi }),\end{eqnarray*}

where the floating covariate, function of time, is defined as *f*(*t*_*π*_) = *β*_4_*t* + *β*_5_*t*^2^ + *β*_6_*t*^3^. The covariate *tested* is the number of tests conducted, *mintemp* is the recorded minimum temperature, and *northwind* is the wind direction. The *northwind* covariate is recorded as a binary variable with north as the reference direction. The reason for that is the wind characteristics of the study area.

In order to improve the case detection rate model in Eq. (10) by introducing the robust logistic regression approach mentioned in Eq. (6) to address the impact outliers, we set the model in Eq. (11) for the case detection probability:

(11) \begin{eqnarray*}{\pi }_{t,s}& =& \gamma \cdot \frac{1}{2} +(1-\gamma )\cdot \text{logit} \left[ \right. {\beta }_{0}+{\beta }_{1}{\text{tested}}_{t,s}+{\beta }_{2}{\text{mintemp}}_{s}\nonumber\\\displaystyle & & +{\beta }_{3}{\text{northwind}}_{s}+I(\cdot )f({t}_{\pi }) \left( \right. .\end{eqnarray*}

In both model components in Eqs. (9) and (11), space index *s* = 0, 1, …, 79 represents the LGAs in Victoria and the time index *t* = 1, 2, …, 73 represents time points from 28 August 2021 to 8 November 2021.

### Model evaluation and implementation

In the initial stage of the model development, diagnostics are reviewed to evaluate the accuracy and representativeness of the Markov Chain Monte Carlo (MCMC) samples and the performance of the parameters. The initial values, thinning, and the number of iterations were re-evaluated to generate the optimal settings for the sub-models and their entirety. After model fitting and diagnostic checking are completed, the goodness-of-fit of the model is reviewed against the observed data.

The data is processed in R software version 3.6.0 (R Core Team, 2019) using several packages. The Bayesian models are fitted using MCMC in JAGS using its feature to support model averaging (Plummer, 2003). In order to incorporate the spatial model, GeoJAGS module is used to generate the structured spatial effect of the model (De Freitas Severino, 2018).

**Figure A1:**
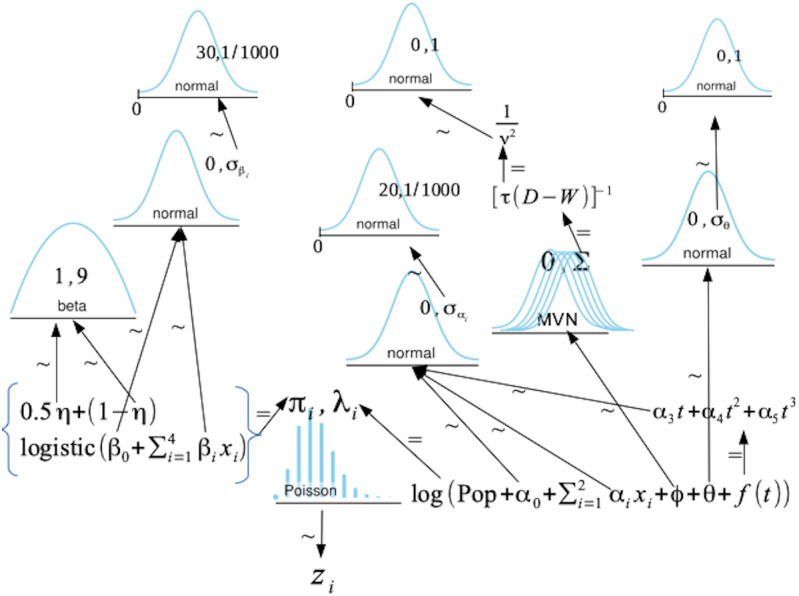
JAGS model diagram of Model 1.

**Figure A2:**
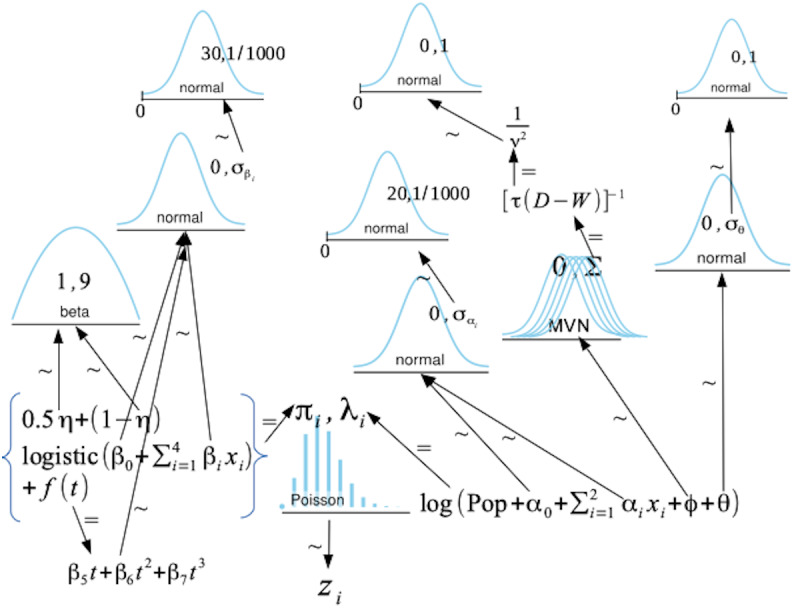
JAGS model diagram of Model 2.

**Figure A3:**
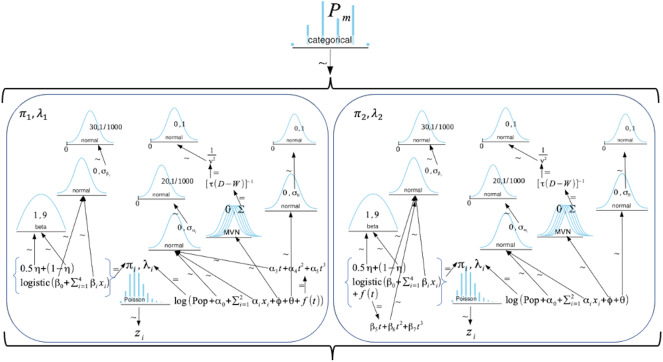
JAGS model diagram of Bayesian model averaging implementation with Models 1 and 2.

Two models are considered in the final run as shown in Table 2. To give parameter estimation and prior specification details, both models’ model diagrams are given in Figs. A1 and A2 of the Appendix. The BMA implementation is outlined in Fig. A3 of the Appendix. The final run of the model in Fig. A3 using JAGS concluded settings of 48,000 iterations, four chains and 11 thinning. We excluded the first 5,000 iterations as a burn-in period. The modes of the generated Markov chains by JAGS for each parameter are taken as the posterior estimates of the parameters.

**Table 2:** Candidate models and their posterior model probabilities.

Model no.	Combination of *λ*_*t*,*s*_ and *π*_*t*,*s*_	*P*(*m*|*z*)
Model 1	*λ*_*t*,*s*_ = pop_*s*_ + *α*_0_ + *α*_1_secondvac_*t*,*s*_ + *α*_2_vaccentres_*s*_ + *ϕ*_*s*_ + *θ*_*s*_	0.001
	*π*_*t*,*s*_ = *β*_0_ + *β*_1_tested_*t*,*s*_ + *β*_2_mintemp_*t*_ + *β*_3_northwind_*t*_ + *f*(*t*_*π*_)	
Model 2	*λ*_*t*,*s*_ = pop_*s*_ + *α*_0_ + *α*_1_secondvac_*t*,*s*_ + *α*_2_vaccentres_*s*_ + *ϕ*_*s*_ + *θ*_*s*_ + *f*(*t*_*λ*_)	0.999
	*π*_*t*,*s*_ = *β*_0_ + *β*_1_tested_*t*,*s*_ + *β*_2_mintemp_*t*_ + *β*_3_northwind_*t*_	

## Results

Table 2 shows the candidate models considered in this study. The framework of this study is two-tiered, which includes the incidence model, *λ*_*t*,*s*_, and the case detection probability model, *π*_*t*,*s*_. The main difference between Model 1 and Model 2 is the categorisation of the floating covariate, the function of time. The models were established to determine whether this floating covariate that is tailored for selected LGAs with a distinct trend pattern should be categorised under the incidence model or the case detection probability model. By letting the data lead to this categorisation, it determines whether the increased number of reported COVID-19 cases is due to increasing incidence in the community or the increased number is driven by the probability of detecting these cases. Alongside this, we also take into account other changes in the environment, such as changes in weather, temperature, and the progression of vaccination rates. In Model 1, the function of time is added in the incidence model *λ*_*t*,*s*_, while in Model 2, it is under the case detection probability model *π*_*t*,*s*_.

**Figure 4:**
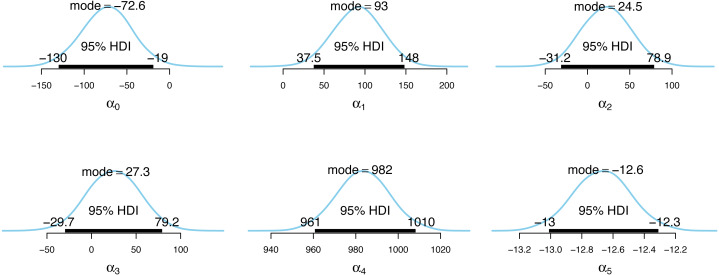
Posterior distributions of the incidence model parameter estimates.

**Figure 5:**
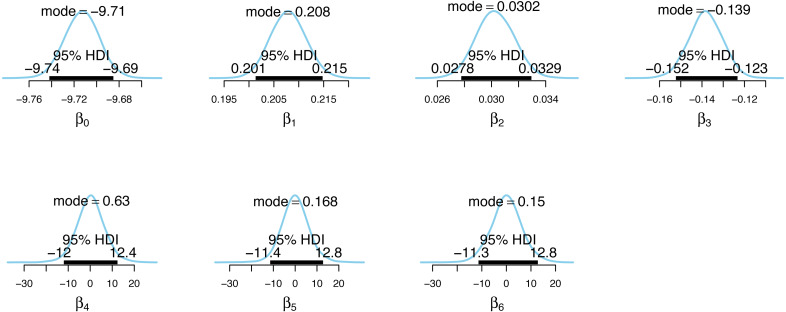
Posterior distributions of the case detection probability model parameter estimates.

The posterior model probabilities, *P*(*m*|*z*), as shown in Table 2, suggest that the function of time for LGAs with a distinct dramatic trend is best explained by the incidence model *λ*_*t*,*s*_ since Model 2 has a significantly larger posterior model probability. The main difference between the models is the inclusion of the floating covariate, which is the function of time. With the two models, we aim to determine whether the 8 LGAs with a drastic increase of daily COVID-19 cases can be explained by ‘the better performance in testing, which detects more cases of COVID-19’ or ‘the areas experienced great and inevitable community transmission leading to more reported COVID-19 cases’ or both. Based on the results of the posterior model probabilities, this drastic increase of COVID-19 cases in the 8 LGAs is explained by the increased community transmission, which means the function of time falls under the incidence component of the model as in Model 2. This is consistent with the significance results of the time predictors in Figs. 4 and 5. This result is a good indication of the importance of vaccination in order to prevent the increase of community transmission in conjunction with other safety measures. Despite the importance of testing, if there is an increasing community transmission where people are experiencing symptoms of COVID-19, this will inevitably surface, and more and more people will get tested. Moreover, during this September outbreak of the pandemic in the state, the highly transmissible and severe disease-causing COVID-19 Delta variant has been around (Australian Government, Department of Health, 2021).

Figs. 4 and 5 show the posterior distributions of the parameters for the incidence model *λ*_*t*,*s*_ and the case detection probability model *π*_*t*,*s*_, respectively. In Fig. 4, the importance of second dose vaccination rates is highlighted by identifying vaccination rates (*α*_1_) as a significant predictor of the COVID-19 daily incidence. From Fig. 5, the testing rates (*β*_1_), minimum temperature (*β*_2_), and wind direction (*β*_3_) are all significant predictors of the case detection probability. Looking at the floating covariate, the function of time *f*(*t*_*λ*_), it clearly reflects the outcome in Table 2 that both the quadratic (*α*_4_) and cubic (*α*_5_) components of the dramatic trends for the selected LGAs is best explained in the incidence model *λ*_*t*,*s*_ while all time components (*β*_4_, *β*_5_, *β*_6_) are insignificant in the case detection probability model *π*_*t*,*s*_.

**Figure 6:**
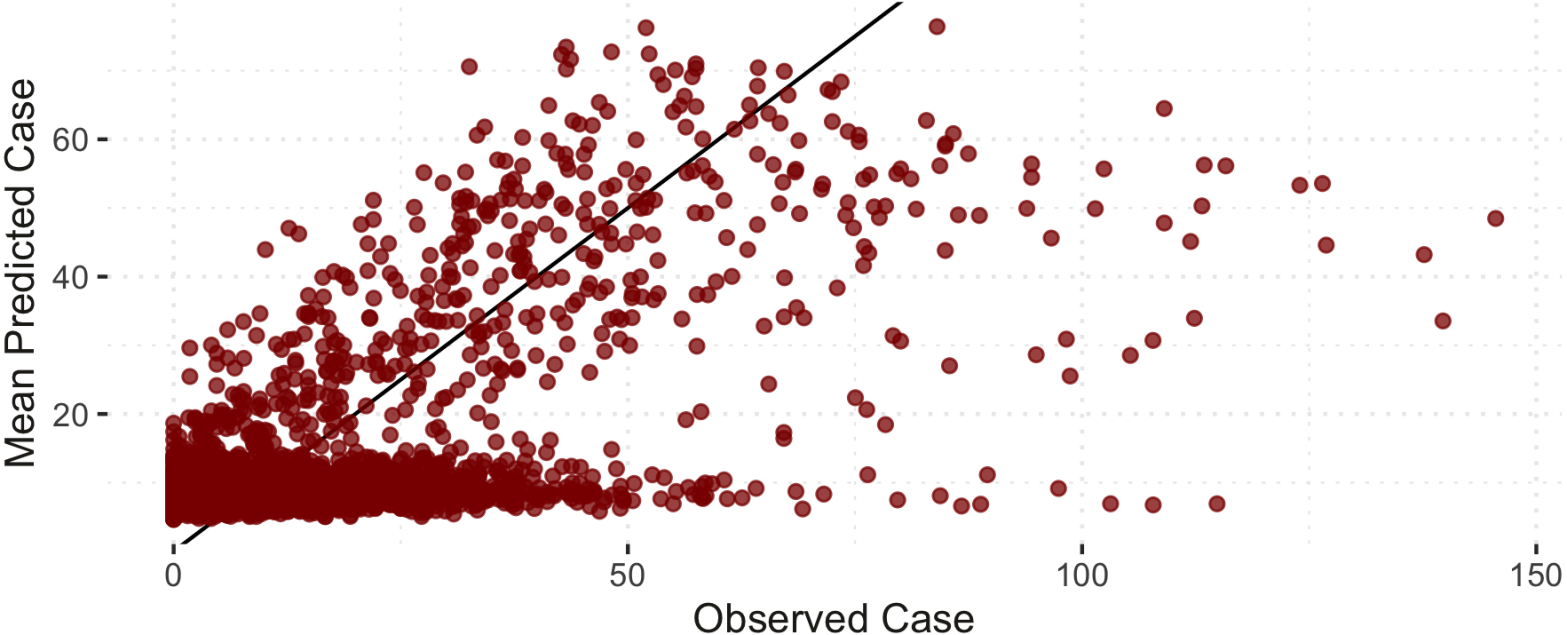
Notified *versus* estimated COVID-19 cases (per 100,000). The black 45-degree line shows the perfect fit for reference.

**Figure 7:**
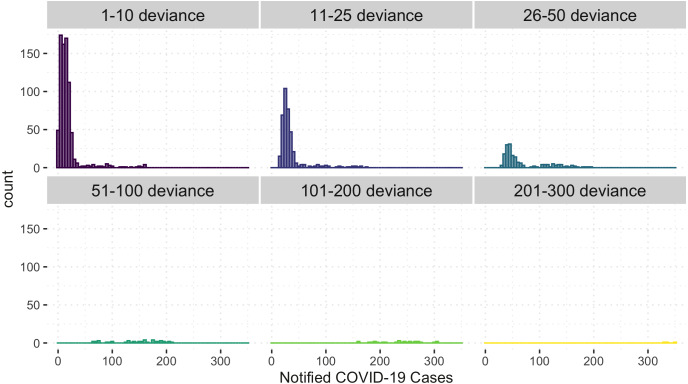
Histogram of under-estimated against notified COVID-19 cases by the model.

Figure 6 shows the comparison of the notified(*reported*) and estimated (*reported + unreported*) COVID-19 cases by the model. This plot helps evaluate the model’s fit against the observed data. The black 45-degrees line shows the perfect fit. The points under this line show under-fitted data points, those above the line are over-fitted data points, and the points on the line are perfectly fitted data points. Therefore, the points above the line are under-reported cases since the predicted number of COVID-19 cases is greater than the reported number of COVID-19 cases. Based on Fig. 6, it is apparent that there is an indication of under-reported cases as represented by points above the line. On the other hand, determining where the model fell short in terms of the points under the line is also of interest. Of note, this study’s daily notified COVID-19 cases are relatively small, ranging from 0 to 350 daily. In order to evaluate the points under the line in Fig. 6, we created a detailed analysis of under-estimated cases by the model in Fig. 7. Figure 7 shows the distribution of the notified COVID-19 cases depending on the deviation of the estimates or the model’s magnitude of the error. Each deviance is grouped accordingly by 1–10, 11–25, 25–50, 51–100, 101–200 and 201–300 points deviation. It shows that most of the cases where the model fell short in the estimates are in the 1-10 deviance range, and these cases have very small notifications ranging from 0-50 daily cases. Our model mostly produces an error margin of 1 to 10 for notified cases less than 25. As the number of notified cases increases, our model’s margin of error dramatically decreases.

**Figure 8:**
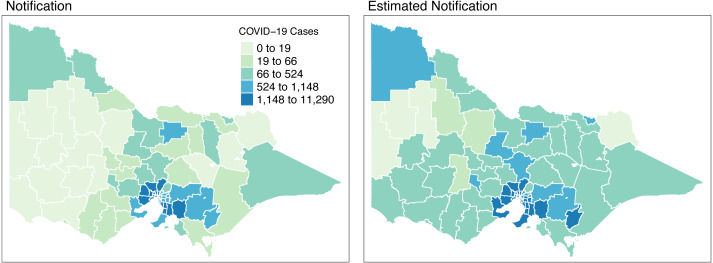
Total distribution of COVID-19 cases in Victoria, Australia from 28 August 2021 to 8 November 2021.

**Figure 9:**
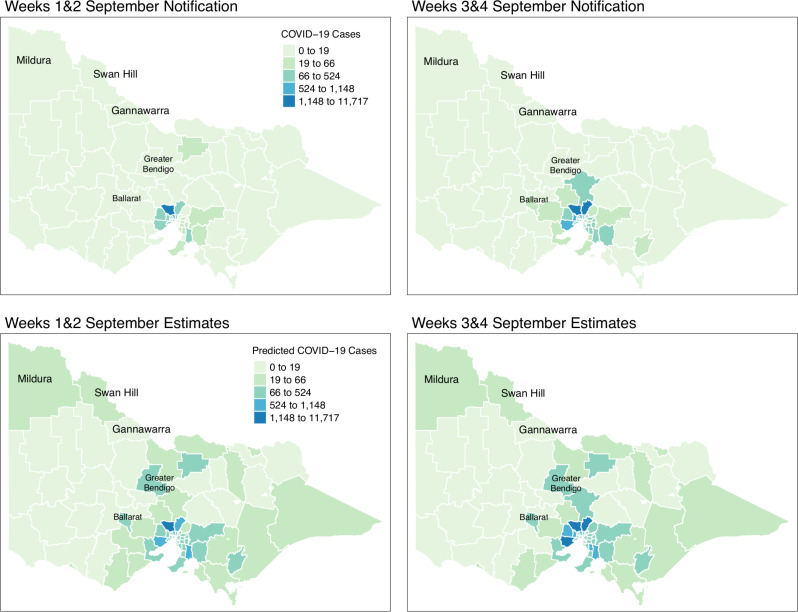
Fortnightly comparison of notified and estimated COVID-19 cases in Victoria, Australia during September 2021.

**Figure 10:**
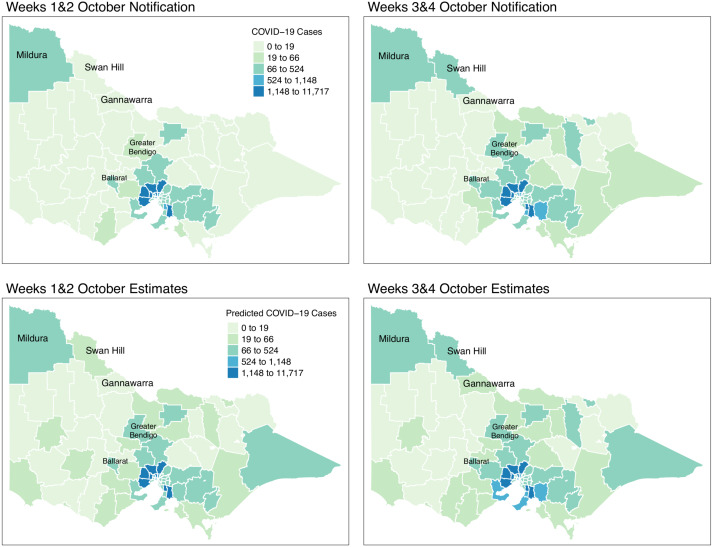
Fortnightly comparison of notified and estimated COVID-19 cases in Victoria, Australia during October 2021.

Figure 8 shows the spatial distribution of the model estimates compared to the notified cases of COVID-19 in Victoria, Australia, from 28 August 2021 to 8 November 2021. This spatial mapping of the estimates shows a good performance of the model fitting to the observed data. Furthermore, it is clear that some areas have under-reported cases as determined by the model. We looked into the spatial estimates on a monthly basis to check any hints of possible under-reporting or outbreak earlier on before it peaked in October. Given this, we spatially mapped the monthly estimates as shown in Figs. 9 and 10.

Figure 9 shows the spatial distribution of the model estimates compared to the state’s notified cases during September 2021. On the other hand, Fig. 10 shows the spatial distribution of the model estimates compared to the notified cases of COVID-19 of the following month, October 2021. In both Figs. 9 and 10, the upper maps are the notified cases of COVID-19 cases on a fortnightly basis while the lower maps are the corresponding estimated cases of COVID-19 cases by our model in the same fortnight.

Remarkably, Figs. 9 and 10 show a progressive detection of COVID-19 cases of the state. Our model was able to show under-reported areas at least two weeks prior to the increase of the notified cases in the area. As an example, in the upper left areas of weeks 1 & 2 and weeks 3 & 4 September notifications, the areas Mildura, Swan Hill, and Gannawarra were showing relatively the same notification counts. Then, in the weeks 1 & 2 and weeks 3 & 4 October notifications, there is a relatively notable increase in these areas. Looking at the estimates for September by our model located in the lower maps of Figs. 9 and 10, early on from weeks 1 & 2 and weeks 3 & 4 September estimates, Mildura, Swan Hill and Gannawarra areas have already been detected to have an increase in the number of COVID-19 cases which leads to the confirmation of the under-reporting in these areas as shown in the weeks 1 & 2 and weeks 3 & 4 October estimates. Using this retrospective analysis of under-reported areas, the model is able to find the missing pieces of a bigger picture of the daily COVID-19 notifications.

We reviewed the results of our model further against the COVID-19 update of Victoria by the Department of Health and Human Services(DHHS). During the pandemic, the Victorian state had been conducting wastewater testing to detect possible cases of COVID-19 cases. According to a DHHS media release dated the 27 of September 2021 (Australian Government, Department of Health and Human Services, 2021), viral fragments were detected in the Ballarat area during 20–22 September. Looking at the notifications during weeks 3 & 4 of September, there is an increase of notifications in that area and further supported by the notifications in October. However, looking at the model estimates early on, weeks 1 & 2 of September, our model has already estimated the increase of COVID-19 cases in the Ballarat area, and it was consistently increasing during the period of the study. The same case is recorded for the Greater Bendigo area. Viral fragments were detected around 21–23 September in the Greater Bendigo area. In weeks 1 & 2 and weeks 3 & 4 of September notifications, the area was showing around 0-19 total fortnightly cases. However, in the model estimates for September, the total number of fortnightly cases is recorded as over 60 cases. These findings show how the model was able to determine the under-reported areas and can be used for early detection, which means more informed planning and surveillance.

## Discussion

Under-reporting is an important issue in disease surveillance to determine the true incidence and burden of disease in addition to strategic planning and intervention. Several studies explored the impact of spatial and temporal differences in the spread of COVID-19 in which inequality in access to testing and other health services is one of the causes of under-reporting. In this study, we proposed using a two-tiered Bayesian model that incorporates the inequalities and interactions between space and time to determine under-reported areas.

Results were able to identify areas with potential under-reporting as shown in Figs. 8, 9 and 10. We reviewed the results at varying time points and locations. Our model was able to detect under-reported cases during weeks 3 & 4 of September, which later surfaced during weeks 1 & 2 of the following month, October. A clear graphical demonstration of these was seen for areas in the state’s upper right side, such as Mildura, Swan Hill and Gannawarra. Moreso, we evaluated the results against the wastewater testing conducted by the state of Victoria. Apparently, the model was able to detect increases in the number of cases beforehand, as shown in areas of Bendigo and Ballarat.

These findings show how the model satisfactorily captured the true incidence of the COVID-19 cases in Victoria using the wide-scope covariates with variations and interactions in space and time, despite the small number of notified COVID-19 cases in the state. This is one of the challenges in data analysis as it becomes harder to accurately model the small number of notified cases. Furthermore, improvements are made in the model compared to the existing framework, such as introducing a floating covariate, Bayesian Model Averaging (BMA), and modification to robust logistic regression of the case detection model to enhance the estimated notifications of the model across space and time.

The availability of the data for the various covariates considered in this study, such as vaccination and testing rates, vaccination and testing centres, wind direction and minimum temperature across spatial locations and time, is a limitation of the study since it is not possible to some of the covariates at all time points and locations. However, the model is very flexible, and other covariates that are more suitable in the disease of interest can be considered for other locations and time periods. In addition, applying this model to other data might require changes to the model covariates to present their spatial and temporal attributes.

## Conclusion

We applied a Bayesian hierarchical framework with Spatio-temporal effects to determine potential under-reporting during the third wave of the COVID-19 pandemic in Victoria, Australia, from 28 August 2021 to 8 November 2021. Findings suggest that under-reported cases in some LGAs of the state turned into local outbreaks before the testing effort reported them. In that sense, our model revealed early detection of increased COVID-19 cases in some LGAs, which were further supported by the wastewater testing results. We also noticed the snowball effect of areas with a high number of reported cases to their contiguous areas with a shared border.

Model-wise, the application of this model is seen to detect the areas that have an increased surge of COVID-19 cases early in addition to areas with a potential under-reporting. Furthermore, our model is applied to Victoria, Australia, where the recorded number of cases is very low, ranging from 0 to 350 cases daily. For statistical models, it becomes harder to get accurate results when the number of reported cases is low. However, our model showed robustness against this issue by producing low levels of deviance. Hence, given the robustness of our model for a small number of COVID-19 cases, we expect this model to perform satisfactorily in datasets with a high recorded number of COVID-19 cases. Moreso, it can confidentially be used for the areas with a low number of reported cases.

The results of this study can help improve the early detection of COVID-19 hotspots and areas with a possibility of under-reporting. These matters are essential in disease surveillance to better design strategies and measures to combat the spread of infectious diseases. In addition, this method is data-driven and can be easily and quickly implemented during an outbreak and in other disease studies.

## Appendix

Figures A1 and A2 are the JAGS model diagrams for the considered model. Figure A3 shows the BMA implementation in JAGS.

## Additional Information and Declarations

### Data Availability

The following information was supplied regarding data availability: The data is available at Zenodo: dinahlope. 2022. dinahlope/Spatiotemporal_Bayesian_COVID19: Added data licences (v1.1.1). Zenodo. https://doi.org/10.5281/zenodo.7042375.
